# The development of lateral flow devices for urinary biomarkers to assess kidney health

**DOI:** 10.1038/s41598-024-59104-5

**Published:** 2024-04-12

**Authors:** Serena D Souza, Wassim Obeid, Jeanine Hernandez, David Hu, Yumeng Wen, Dennis G. Moledina, Andre Albert, Anya Gregg, Andrew Wheeler, Heather Thiessen Philbrook, Chirag R. Parikh

**Affiliations:** 1 Division of Nephrology, Department of Medicine, Johns Hopkins University School of Medicine, 1830 E. Monument St., Suite 416, Baltimore, MD 21287 USA; 2 Section of Nephrology and Clinical and Translational Research Accelerator, Department of Internal Medicine, School of Medicine, Yale University, New Haven, CT USA; 3 Mologic Inc (D/B/A Global Access Diagnostics), 83 Pineland Drive, Gray Hall Suite 202, New Gloucester, ME USA; 4 Mologic Ltd (D/B/A Global Access Diagnostics), Bedford Technology Park, Thurleigh, UK

**Keywords:** Biomarkers, Nephrology

## Abstract

Serum creatinine levels are insensitive to real-time changes in kidney function or injury. There is a growing interest in assessing kidney injury by measuring biomarkers in body fluid. From our previous studies, we identified and reported three urinary biomarkers namely Uromodulin (UMOD), Osteopontin (OPN), and Interleukin-9 (IL-9) to be associated with kidney health. The availability of a rapid point-of-care test for these urinary biomarkers will potentially accelerate its applicability and accessibility. In this study, we aimed to develop novel lateral flow device (LFD) for UMOD, OPN and IL-9. We tested paired antibodies using Enzyme Linked Immunosorbent Assay wherein we observed functionality only for UMOD and OPN and not for IL-9. A conjugation buffer pH of 7.8 and 8.5 was found suitable at a detection antibody concentration of 15 µg/mL for LFD development. The developed LFDs were found to quantitatively measure UMOD standard (LLOD of 80,000 pg/mL) and OPN standard (LLOD of 8600 pg/mL) respectively. The LFD was also able to measure human urinary UMOD and OPN with a percent CV of 12.12 and 5.23 respectively.

## Introduction

Serum creatinine (SCr) has been used as an established routine test for diagnosing kidney disease for decades. Elevated SCr levels constitute a determining parameter for diagnosis of acute kidney injury (AKI)^1^, chronic kidney disease (CKD)^2^, in treatment procedures such as dialysis^3^, and organ allocation for transplantation as part of the Kidney Donor Profile Index (KDPI) score^4^. Despite the importance of creatinine measurement in medical decision-making, creatinine is an indirect marker of kidney damage with poor sensitivity to real-time changes that occur in kidney tissue during injury^5^. This highlights the need to develop and utilize biomarker(s) that can provide relevant information about the health of the kidney in question. Kidney repair and injury biomarkers are gaining steady importance in research and clinical applicability in conjunction with existing tests due to the real-time information they provide. Uromodulin (UMOD), the most abundant protein in the urine, indirectly measures nephron mass and kidney function^6^. Mutations in the gene encoding UMOD believed to cause autosomal dominant tubulointerstitial kidney disease (ADTKD) are reported to be a common cause of CKD^7^. Serum UMOD levels were found to be a strong predictor of graft loss in renal transplant recipients^8^. Osteopontin (OPN) was found to play a protective role in kidney stone formation^9^ and in acute renal allograft rejection^10^. Our study reported a low ratio of UMOD to OPN to predict favorable kidney graft outcomes in deceased donor urine samples^11^. Interleukin-9 (IL-9) has been gaining momentum in its beneficial role in glomerulonephritis^12^ and ischemia–reperfusion injury^13^. Our studies on IL-9 have highlighted its diagnostic and prognostic role in acute interstitial nephritis^14,15^. These findings point towards the possible use of UMOD, OPN and IL-9 in assessing the quality of kidneys in several disease conditions such as kidney transplant and acute interstitial nephritis.

Along with research focused on identifying predictive biomarkers, it is important to ensure the feasibility and efficiency of these tests in real-world scenarios. Existing techniques such as conventional enzyme-linked immunosorbent assay (ELISA) and MesoScale Discovery (MSD) require high-end infrastructure in a laboratory setting and need to be executed by skilled personnel. While the latter serves as a modus operandi, there is an echoing need felt for the availability of testing procedures that can be performed at the Point-of-Use or Point-of-Care *such as* lateral flow devices (LFDs). The field of nephrology is no stranger to the world of Point-of-Care technologies. Dipsticks have been used for the screening of kidney disease and routine urine analysis of albumin, blood, and infection^16^. Test strips with chemicals impregnated onto them are used to detect a range of analytes including glucose, bilirubin, pH etc*.* which have been marketed and widely used across the globe, indicating a huge potential for the applicability and usability of LFDs in this domain.

In our study, we pursued development of a sandwich ELISA based lateral flow device using gold nanoparticles for the detection of the identified urinary biomarker(s) (UMOD, OPN and IL-9).

## Results

**Figure 1:**
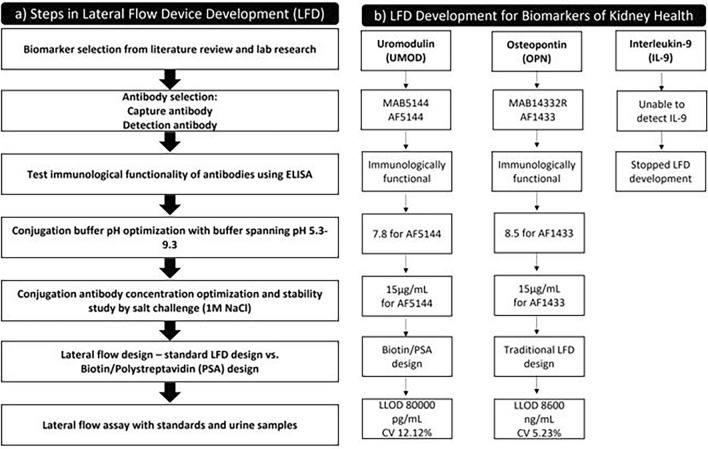
Approach employed in lateral flow device development. (**a**) Steps involved in lateral flow device development (LFD); (**b**) LFD development for biomarkers of kidney health.

The biomarkers UMOD, OPN, IL-9 were selected after a thorough review of literature along with the results of the research carried out by our research team^11,15^. The antibodies were procured based on the company's recommendation (R and D systems). The approach adopted to transfer selected biomarkers onto the lateral flow device is summarized in Fig. 1a,b and described below.

### Immunological testing of the antibody conjugates using ELISA

**Figure 2:**
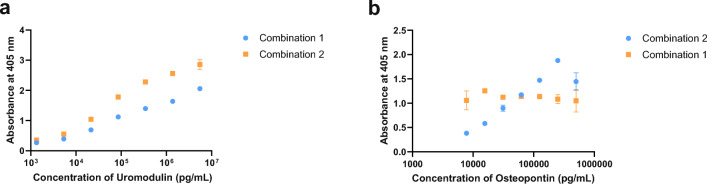
Antibody pair screening using Enzyme Linked Immunosorbent Assay (ELISA). (**a**) Uromodulin ELISA: Combination 1: Capture antibody AF5144, Detection antibody MAB5144; Combination 2: Capture antibody MAB5144; Detection antibody AF5144. (**b**) Osteopontin ELISA: Combination 1: Capture antibody AF1433, Detection antibody MAB14332R; Combination 2: Capture antibody MAB14332R; Detection antibody AF1433.

Of the three antibody pairs tested for UMOD, OPN and IL-9 respectively, the antibody pair for only UMOD was found to be immunologically functional. Although the use of either antibody pair as detection antibody or capture antibody was able to detect UMOD effectively, the combination of MAB5144 as capture antibody and AF5144 as detection antibody provided greater sensitivity and was chosen for further studies (Fig. 2a; Table 1). The antibody pairs for OPN (AF1433, MAB1433) and IL-9 (AF209, MAB209) were unable to detect the respective antigens satisfactorily (Supplementary Table S1). Additional antibodies were tested for OPN and IL-9 antigens. In this second attempt, the modified antibody pair (MAB14332R as capture antibody, AF1433 as detection antibody) for OPN was found to be immunologically functional and was used for further studies (Fig. 2b; Table 1). The modified IL-9 antibody pair (MAB2091, AF209) was unable to detect IL-9 in either combination of capture antibody or detection antibody in ELISA (Supplementary Table S1). Given these results, further work for lateral flow design was carried out with only UMOD and OPN.

**Table 1:** Paired antibody confirmed with immunological functionality using Enzyme Linked Immunosorbent Assay (ELISA) for Uromodulin (UMOD) and Osteopontin (OPN).

Uromodulin (UMOD)			Osteopontin (OPN)	
Standard (pg/mL)	Combination #1 AF5144 (Capture Ab) MAB5144 (Detection Ab)	Combination #2 MAB5144 (Capture Ab) AF5144 (Detection Ab)	Standard (pg/mL)	Combination #2 MAB14332R (Capture Ab) AF1433 (Detection Ab)
	Mean absorbance (SD)	Mean absorbance (SD)		Mean absorbance (SD)
5,500,000	2.060 (0.042)	2.860 (0.164)	500,000	1.445 (0.182)
1,375,000	1.639 (0.063)	2.563 (0.003)	250,000	1.878 (0.043)
343,750	1.398 (0.045)	2.281 (0.048)	125,000	1.471 (0.040)
85,938	1.121 (0.031)	1.782 (0.010)	62,500	1.171 (0.041)
21,484	0.693 (0.049)	1.041 (0.004)	31,250	0.897 (0.062)
5371	0.391 (0.035)	0.551 (0.004)	15,625	0.581 (0.020)
1343	0.269 (0.011)	0.357 (0.001)	7812.5	0.382 (0.033)
0	0.241 (0.021)	0.290 (0.01)	0	0.095 (0.002)

### Conjugation buffer pH optimization and conjugation concentration optimization

The optimum conjugation pH was found to be 7.8 for AF5144 (UMOD) and 8.5 for AF1433 (OPN) respectively (Table 2). The concentration of antibody needed for conjugation and the stability of the gold antibody conjugate was assessed by performing a salt challenge. Concentrated sodium chloride solution causes the gold conjugate to become unstable and aggregate if it has not conjugated to the antibodies stably. An antibody loading concentration of 15 µg/mL was found to be sufficient to form stable gold conjugate for AF5144 (UMOD) and AF1433 (OPN) post salt challenge (Table 3). These optimized conditions were further used to develop the lateral flow designs.

**Table 2:** Conjugation buffer pH optimization.

Conjugation buffer pH	AF5144 (Detection antibody) for Uromodulin (UMOD)	AF1433 (Detection antibody) for Osteopontin (OPN)
	Absorbance ratio (550/600)	Absorbance ratio (550/600)
9.3	4.16	4.18
9	4.14	4.07
8.5	4.38	4.36 (optimal)
7.8	4.40 (optimal)	4.16
7.5	4.31	4.32
7.1	4.12	3.46
6.4	3.78	3.07
5.3	1.97	1.02

**Table 3:** Conjugation antibody concentration optimization with salt challenge.

Antibody loading concentration (µg/mL)	AF5144 (Detection antibody) for Uromodulin (UMOD)	AF1433 (Detection antibody) for Osteopontin (OPN)
	Absorbance ratio (550/600)	Absorbance ratio (550/600)
0	1.2	1.2
5	0.9	1.0
10	2.8	2.7
15	4.2	4.0
20	4.4	4.1
25	4.0	4.1
30	4.4	4.3

### Lateral flow device development and testing using UMOD/OPN standards and urine samples

**Figure 3:**
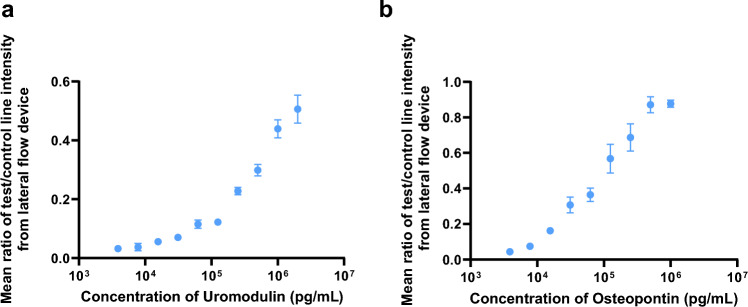
Standard curve developed for (**a**) UMOD using Biotin/Polystreptavidin based Lateral Flow Device and (**b**) OPN using standard Lateral Flow Device. Three drops of UMOD standard (0–2,000,000 pg/mL) and OPN (0–1,000,000 pg/mL) were run on biotin/polystreptavidin (PSA)/standard lateral flow device respectively for 20 min and the intensity of control line and test line was read using RDS- 2500 reader (DETEKT, USA). The mean ratio of the Test line/ Control line from the LFD was computed, and a semi-log plot was generated to obtain a standard curve. Data is represented as Mean ratio of Test line/Control line intensity (SD).

For the UMOD LFD, the standard LFD design was developed wherein the capture antibody formed the test line by passive adsorption, a generic IgG antibody constituted the control line, and the gold conjugated antibody was deposited onto the conjugate pad. The test strips were tested by running phosphate-buffered saline—Tween 20 (PBST 0.1%) to check for non-specific binding (NSB). A high NSB was observed for the UMOD lateral flow strip with the buffer itself. The use of 1% BSA as a blocking agent was not found to ameliorate the issue. To overcome this challenge for UMOD, a biotin/polystreptavidin (PSA) format was used to design the lateral flow strips. This change was found to alleviate the issue of NSB for UMOD and was used for further experiments. (Supplementary Table S2). A concentration-dependent response was observed for UMOD standard with a LLOD of 80,000 pg/mL (Fig. 3a; Supplementary Table S3).

A hook effect was observed when OPN standard prepared in PBST 0.1% buffer was used at concentration greater than 250,000 pg/mL (Supplementary Table S4). It was found that reducing the capture antibody concentration from 1 to 0.5 mg/mL overcame the issue of NSB. This modified design was used for further studies. A concentration dependent response was observed for OPN standard with a LLOD of 8600 pg/mL (Fig. 3b; Supplementary Table S3). The LFD was able to reliably measure human urinary UMOD and OPN with a percent CV of 12.12 and 5.23 respectively (Table 4).

**Table 4:** Lateral flow device testing with human urine samples.

S. no	UMOD			OPN		
	Mean test line intensity (SD)	Mean control line intensity (SD)	Mean ratio of test line/control line	Mean test line intensity (SD)	Mean control line intensity (SD)	Mean ratio of test line/control line
1	549,597.5 (85,204.25)	1,020,824 (17,962.63)	0.54	423,211 (77,614.87)	623,625.5 (58,131.96)	0.68
2	416,851.5 (46,176.19)	881,810 (23,218.56)	0.47	438,677.5 (28,149.21)	590,046 (64,393.39)	0.74
3	304,018.5 (64,051.85)	613,022 (60,258.23)	0.50	183,160.5 (21,134.71)	624,369.5 (59,168.57)	0.29
4	383,004.5 (18,842.27)	618,569 (99,784.08)	0.62	456,867 (220,477.31)	495,898 (220,774.29)	0.92
5	302,050.5 (78,560.27)	463,271.5 (179,119.34)	0.65	507,758.5 (104,621.40)	594,838.5 (153,397.62)	0.85

## Discussion

There is an urgent need to develop approaches and tools which can assess the quality and health of the kidney in real time to accurately and efficiently ascertain kidney disease. Urinary proteins such as UMOD, OPN and IL-9 could serve as credible biomarkers to supplement existing clinical tests. UMOD, synthesized by the renal tubular epithelial cells and a major component of hyaline casts, is known to aid kidney health^17,18^. UMOD has also been used as a marker for ADTKD^7^. OPN, synthesized in the thick ascending limb, T-cells^19^ among others, is reported to play a key role in kidney physiology^20^. The combined use of the levels of UMOD and OPN in deceased donors as a ratio has been reported to provide valuable information regarding kidney graft outcomes^11^. IL-9, a pro-inflammatory cytokine^21^, was reported to aid discrimination in AIN diagnosis and predict kidney function^14,14^. IL-9 was recently shown to have a renal release post kidney transplantation in brain dead donors^22^. The aforementioned biomarkers were chosen in our study as a possible panel to assess kidney quality in deceased donors in kidney transplantation in particular.

The antibody pairs tested for IL-9 were unable to detect IL-9 in a concentration dependent manner in the range of interest as observed from the ELISA results. This illustrates the importance of experimentally screening antibody pairs to check functionality using suitable techniques such as ELISA prior to LFD development. We developed quantitative LFDs for the urinary biomarkers UMOD and OPN, which are able to estimate reliable biomarker levels in both standard and human urine samples.

There is no other reported study to the best of our knowledge which has focused on developing either a qualitative or quantitative LFD for UMOD and OPN. However, there have been several studies focusing on the development of Point-of-Care solutions to support kidney health such as CysC, a marker used to estimate glomerular filtration rate. Natarajan et al.^23^, developed a fluorescence-based lateral flow assay using a monoclonal antibody-based sandwich ELISA whereas Chen et al.^24^ provided a proof-of-concept LFA to estimate CysC in skin interstitial fluid using a hydrogel-based microneedle patch integrated LFA. These studies are similar to our study with respect to the design of LFAs and the immunocomplex design used for the estimation of the analyte and differ in the detection system and sample collection apparatus respectively. Zhang et al.^25^, demonstrated the detection of CysC using both gold nanoparticles and gold nanorods based LFA to improve the colorimetric sensitivity. Selier et al.^26^ devised an antibody aptamer hybrid LFA assay for detecting CXCL9, a marker indicative of antibody-mediated rejection in kidney transplantation recipients. The combinatorial use of nanoparticles or nanorods and antibody aptamer hybrid system provides insight into alternative ways of improving detection capabilities of the LFA.

Our study has several strengths. First, the meticulous immunological testing of the chosen antibodies helped in identifying the best antibody to be used for colloidal gold conjugation. The need for testing additional antibodies for OPN pointed out the necessity of validating the functionality of antibodies prior to their use in LFD development. Second, the change in the LFD design for UMOD from standard to biotin and PSA format as compared to OPN provided an insight regarding the need for the custom development of LFDs for each biomarker of interest. Additionally, the antibody AF5144 used for the UMOD ELISA and LFD has been reported to recognize both polymeric and non-polymeric forms in human urine thereby providing total uromodulin measurements^27^. Third, the developed LFDs enabled the quantitative estimation of UMOD and OPN across a large concentration range which can better help understand kidney disease. The limitations of this study are firstly, the developed LFD is a multi-step test requiring centrifugation and dilution of the sample prior to use thereby requiring additional instrumentation, and training to perform the test. Second, the incubation time for the LFD readout is currently 20 min and may be not rapid enough in certain settings. Third, the LFD for UMOD and OPN are currently developed as two separate LFD’s and improvements could be made to develop a single LFD which can quantitatively estimate both antigens on one LFD.

## Conclusion

Immunological testing of antibodies along with conjugation-stability studies were found to be the foundation of an effective LFD. The design of the LFD was found to be biomarker dependent and played a crucial role in improving the sensitivity of the assay. A combination of these factors led to the development of the Biotin/PSA LFD and a standard format LFD for quantitative estimation of UMOD and OPN standards and in human urine samples. Future studies should study additional performance parameters of the LFD and evaluate the performance of these biomarker LFDs in clinical settings to aid medical decisions.

## Materials and methods

Phosphate buffered saline (PBS) (Sigma P4417-199TAB), Tris (50 mM, Sigma T6066) buffered saline (150 mM, Fisher Scientific S/3161/65) with Tween-20 (0.01%, Sigma P1379) (TBST) were prepared and used for the study. Microtiter ELISA plates were procured from Costar 9018, Thermo Fisher Scientific whereas the plate reader and RDS-2500 reader were purchased from FLUOstar Omega Microplate Reader (BMG Labtech) and (DETEKT, USA) respectively. Anti-species alkaline phosphatase (A5187 and SAB3700286) was procured from Sigma, USA. The substrate pNPP was procured from BioPanda, USA. The paired antibodies used in the study are summarized in Table 5.

**Table 5:** Paired antibody and recombinant protein standard used for the study.

	Antibody	Recombinant protein standard
UMOD	MAB5144; AF5144 (R&D Systems, USA)	H00007369-Q01 (Novus Biologicals, USA)
OPN	MAB1433; MAB14332R; AF1433 (R&D Systems, USA)	1433-OP (R&D Systems, USA)
IL-9	MAB2091; AF209; MAB209 (R&D Systems, USA)	209-ILB010 (R&D Systems, USA)

### Immunological testing of the antibody using ELISA:

The microtiter ELISA plates (Costar 9018, Thermo Fisher Scientific) were coated with 100 µL of respective coating antibody at a concentration of 0.5/1 µg/mL in PBS and incubated overnight at room temperature. The plates were washed thrice with 300 µL TBST using a plate washer (BioTek 405). The wells were blocked with 150 µL PBST 0.1% BSA 1% and incubated at room temperature for 1 h on a shaker. The blocking solution was discarded, the plates were washed thrice with 300 µL TBST and incubated at room temperature with respective 100 µL antigen for 1 h on shaker. After discarding the antigen solution, the plates were washed thrice with 300 µL TBST and incubated with the respective 100 µL detection antibody (0.5/1 µg/mL in PBST 0.1% BSA 1%) for 1 h at room temperature on shaker. Post incubation, the antibody solution was discarded and following a final wash, the plates were incubated at room temperature with 100 µL of respective anti-species alkaline phosphatase at a dilution of 1:30 k/1: 10 k for 1 h on shaker at room temperature. The plates were incubated at room temperature in the dark with 100 µL pNPP and the absorbance was read at 405 nm after 30/45 min depending on the assay.

### Conjugation buffer pH optimization and conjugation concentration optimization

1.5 µL of the detection antibody (1 mg/mL) was mixed with 5 µL of conjugation buffer (pH spanning from 5.3 to 9.3—20 mM MES pH 5.3, 20 mM BES pH6.4, 20 mM TES pH7.1 and 7.5, 20 mM TAPS pH 7.8 and 8.5, 20 mM Borate pH 9.0 and 9.3) and 0.1 mL colloidal gold (EM.GC40, BBI Solutions) in microtiter ELISA wells (StarLab, E2996-1600) on a plate shaker for 10 min. The absorbance was read at 530 nm, 550 nm and 600 nm on FLUOstar Omega Microplate Reader (BMG Labtech). The aggregation ratio of Abs 550/600 was calculated. To determine the antibody loading concentration, 5 µL of the selected buffer was mixed with varying volumes of the antibody solution (1 mg/mL) to obtain a loading concentration of 0–30 µg/mL. To this mixture, 100 µL of colloidal gold was added and incubated for 10 min. The mixture was subjected to a salt challenge by adding 10 µL of 1 M NaCl. The absorbance was read at 530, 550 and 600 nm on FLUOstar Omega Microplate Reader (BMG Labtech). The aggregation ratio of Abs 550/600 was calculated.

### Lateral flow strip development

The lateral flow test strip consisted of a sample pad (Ahlstrom-Munksjo, USA, Product 8964)**,** conjugate pad (Ahlstrom-Munksjo, USA, Product 8964), CN140 nitrocellulose test membrane (Sartorius, 1UN14ER100025NT USA) and a wicking adsorbent pad (Ahlstrom-Munksjo, USA, Product 222) onto a backing card (Kenosha, Netherlands, product KN-2211). For the UMOD assay, a biotin—polystreptavidin (PSA) format was used to design the lateral flow strip. The test and control lines were prepared by spraying (1 mg/mL polystreptavidin-R, BioTez, Berlin). The membranes were incubated at a temperature of 50 °C for 5 min. The membranes were dried at room temperature in a desiccator to remove excess moisture. The LFD was assembled and stored until further use.

For the OPN assay, the test strips with capture antibody at a concentration of 0.5 mg/mL were prepared by mounting the CN140 nitrocellulose membrane (Sartorius, 1UN14ER100025NT USA) onto a backing card (Kenosha, Netherlands, product KN-2211) with a conjugate pad (Ahlstrom-Munksjo, USA, Product 8964) containing the detection antibody (15 µg load level, 5 OD/device) sprayed down as a gold conjugate. The membranes were incubated for 5 min at 50 °C to remove most of the moisture and further dried at room temperature in a desiccator. The LFD was assembled and stored until further use.

### Lateral flow device (LFD) testing

#### Using recombinant protein standards

**Figure 4:**
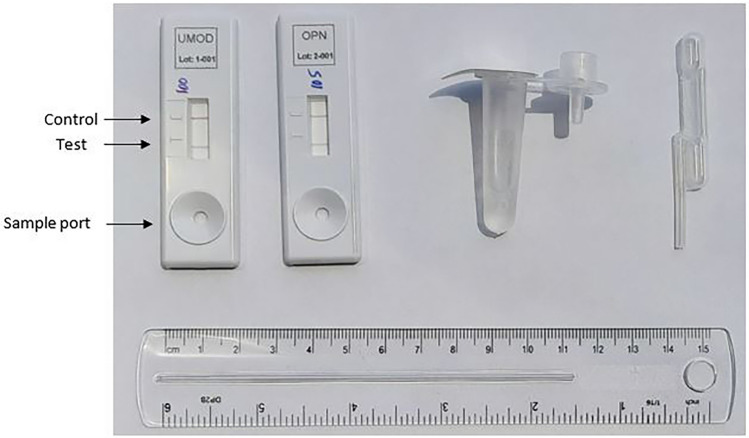
Lateral flow device assembly kit for urinary biomarkers Uromodulin (UMOD) and Osteopontin (OPN). (Left to Right) Biotin/Polystreptavidin (PSA) based LFD for UMOD, Standard LFD for OPN, Diluent tube and Disposable pipette.

A recombinant protein standard curve (hereafter referred to as standard) was generated for UMOD (0–2,000,000 pg/mL) and for OPN (0–1,000,000 pg/mL) on a biotin/polystreptavidin based lateral flow device and standard lateral flow device for UMOD and OPN respectively. The UMOD and OPN standards were prepared and filled in a custom-made diluent tube (Fig. 4). Three drops of the respective standards were added to the devices and incubated on a flat surface for 20 min at room temperature. The results were read using a handheld RDS-2500 reader (DETEKT, USA) in the form of control line (CL) and test line (TL) intensity. A ratio of the TL/CL intensity was computed to analyze the results.

#### Human urine samples

Human urine samples (n = 5) were collected from deceased organ donors and tested for UMOD and OPN in duplicates. All methods were carried out in accordance with relevant guidelines and regulations. All experimental protocols were approved by Johns Hopkins Institutional Review Board (IRB no: IRB00248332). Informed consent was obtained from next of kin and family. For the measurement, the urine samples were centrifuged at 2000 rpm for 12 min. 50 µL of the centrifuged sample was pipetted using a custom-made pipette and added to the diluent in the custom-made diluent tube. The diluent tube was inverted 5 times. Three drops of this diluted sample were added to the LFD and incubated for 20 min before being read on the RDS—2500 reader.

### Supplementary Information

Supplementary Tables.

## Data Availability

All data generated or analyzed during this study are included in this published article (and its Supplementary Information files).
