# Supplementary material for: The development of lateral flow devices for urinary biomarkers to assess kidney health
Source: Sci Rep. 2024 Apr 12;14:8516. doi: 10.1038/s41598-024-59104-5 (PMC11014899; doi:10.1038/s41598-024-59104-5)
Supplement: Supplementary file 1 — Supplementary Tables. [file 41598_2024_59104_MOESM1_ESM.pdf]

The development of lateral flow devices for urinary biomarkers to assess kidney health

Serena D Souza[1], Wassim Obeid[1], Jeanine Hernandez[1], David Hu[1], Yumeng Wen[1], Dennis G. Moledina[2], Andre Albert[3], Anya Gregg[4], Andrew Wheeler[3], Heather Thiessen Philbrook[1] and Chirag R Parikh[1]*

1: Division of Nephrology, Department of Medicine, Johns Hopkins University School of Medicine, Baltimore, MD

2: Section of Nephrology and Clinical and Translational Research Accelerator, Department of Internal Medicine, School of Medicine, Yale University, New Haven, CT

3: Mologic Inc (D/B/A Global Access Diagnostics), 83 Pineland Drive, Gray Hall Suite 202, New Gloucester, ME

4: Mologic Ltd (D/B/A Global Access Diagnostics), Bedford Technology Park, Thurleigh, UK

Correspondence:

Dr Chirag Parikh

Director, Division of Nephrology

Johns Hopkins University School of Medicine

1830 E. Monument St., Suite 416, Baltimore, MD 21287

Phone: (410) 955-5268

Email: chirag.parikh@jhmi.edu

Supplementary Table S1: Antibody pair screening using Enzyme Linked Immunosorbent Assay (ELISA)

**Osteopontin (OPN)**					**Interleukin-9 (IL-9)**					
**Standard (pg/mL)**	**Combination #1<br><br>AF1433 (Capture Ab) MAB1433 (Detection Ab)**	**Combination #2<br><br>MAB1433 (Capture Ab) AF1433 (Detection Ab)**	**Standard (pg/mL)**	**Combination #1 AF1433 (Capture Ab) MAB14332R (Detection Ab)**	**Standard (pg/mL)**	**Combination #1<br><br>AF209 (Capture Ab) MAB209 (Detection Ab)**	**Combination #2<br><br>MAB209 (Capture Ab) AF209 (Detection Ab)**	**Standard (pg/mL)**	**Combination #1<br><br>AF209 (Capture Ab) MAB2091 (Detection Ab)**	**Combination #2<br><br>MAB2091 (Capture Ab) AF209 (Detection Ab)**
	Mean Absorbance (SD)			Mean Absorbance (SD)		Mean Absorbance (SD)			Mean Absorbance (SD)	
**2,90,000**	0.077 (0.002)	0.215 (0.003)	**5,00,000**	1.052 (0.231)	**2109**	0.067 (0.003)	0.187 (0.002)	**1,00,000**	0.430 (0.003)	0.405 (0.042)
**58,000**	0.081 (0.001)	0.198 (0.004)	**2,50,000**	1.084 (0.090)	**421.8**	0.068 (0.003)	0.197 (0.001)	**1,000**	0.093 (0.0007)	0.095 (0.003)
**11,600**	0.082 (0.001)	0.189 (0.007)	**1,25,000**	1.136 (0.013)	**84.39**	0.067 (0)	0.194 (0.001)	**500**	0.091 (0.0007)	0.098 (0.009)
**2,320**	0.084 (0.001)	0.179 (0.002)	**62,500**	1.140 (0.015)	**16.87**	0.075 (0.005)	0.193 (0.006)	**250**	0.090 (0)	0.090 (0)
**464**	0.083 (0.001)	0.186 (0.006)	**31,250**	1.122 (0.003)	**3.374**	0.073 (0.001)	0.195 (0.011)	**125**	0.090 (0.0007)	0.088 (0)
**92.8**	0.088 (0.002)	0.202 (0.004)	**15,625**	1.256 (0.015)	**0.675**	0.078 (0.004)	0.210 (0.001)	**62.5**	0.089 (0)	0.091 (0.0007)
**18.56**	0.093 (0.006)	0.210 (0.003)	**7,812.5**	1.058 (0.193)	**0.135**	0.074 (0.001)	0.207 (0.003)	**31.25**	0.099 (0.012)	0.088 (0)
**0**	0.089 (0.001)	0.209 (0.012)	**0**	1.164 (0.290)	**0**	0.076 (0.004)	0.199 (0.012)	**15.625**	0.093 (0.005)	0.089 (0.0007)
								**0**	0.090 (0.002)	0.091 (0.003)

Supplementary Table S2: Lateral flow device format development for Uromodulin (UMOD)

**Antigen**	**Lateral flow device format**	**Capture Antibody**	**Detection Antibody**	**Buffer only**		**Readout interpretation**
				PBST	PBST 1%BSA	The intensity was read using Optricon 'Cube' reader.<br>Cube reader value greater than 10 units indicates a visible line.
UMOD	Standard	MAB5144	AF5144	97	132	
	Biotin/Polystreptavidin	MAB5144	AF5144	10<br>9.1	-	

Supplementary Table S3: Lateral flow device testing with Uromodulin (UMOD) and Osteopontin (OPN) standards

	**Uromodulin (UMOD)**			**Osteopontin (OPN)**		
**Concentration (pg/mL)**	**Mean Test line intensity (SD)**	**Mean Control line intensity (SD)**	**Mean ratio of test line/ control line (SD)**	**Mean Test line intensity (SD)**	**Mean Control line intensity (SD)**	**Mean ratio of test line/ control line (SD)**
**2,000,000**	451955 (66410.84)	894104.7 (109756.3)	0.505	-	-	-
**1,000,000**	433564.5 (27145.12)	986888 (6829.237)	0.440	685593 (43253.7)	782722 (67559.81)	0.876
**5,00,000**	322426.7 (36789.05)	1076556.3 (52215.88)	0.299	719718.67 (16073.8)	826994.67 (24603.16)	0.870
**2,50,000**	210401.5 (33786.27)	921539 (96869.39)	0.228	581651.67 (72818.1)	845309.33 (23679.50)	0.688
**1,25,000**	106642 (22949.86)	868100 (120424.5)	0.123	447241.67 (58556.6)	789090.67 (49094.37)	0.567
**62,500**	105083.7 (10957.44)	922994.3 (167329.2)	0.114	344838.5 (27220.1)	947460 (23200.17)	0.364
**31,250**	67714.33 (10218.75)	964041.3 (136933.3)	0.070	299167.67 (44590.9)	975798.33 (45438.55)	0.306
**15,625**	56787.67 (11307.86)	1014721 (88147.82)	0.056	134895.33 (11465.2)	835440 (69016.25)	0.161
**7,810**	39745.5 (13356.54)	1062762.5 (9873.332)	0.037	60605.5 (5326.64)	810664 (6916.92)	0.075
**3,900**	31883.33 (3240.635)	998204 (65509.52)	0.032	36607.5 (6387.3)	826905.5 (113038.80)	0.044
**0**	57228.33 (19217.75)	1053163 (134867.9)	0.054	7427.33 (635.297)	963014 (74980.03)	0.008
Intensities were read using RDS-2500 reader (DETEKT, USA)						

Supplementary Table S4: Lateral flow device development data for Osteopontin (OPN) when capture antibody was coated at 1 mg/mL concentration

**Osteopontin standard curve (pg/mL)**	**Replicate 1**	**Replicate 2**	**Average**	**Readout interpretation**
10,000,000	66	69	67.5	The intensity was read using Optricon 'Cube' reader.<br>Cube reader value greater than 10 units indicates a visible line.
1,000,000	171	163	167	
5,00,000	180	197	188.5	
2,50,000	196	188	192	
50,000	165	155	160	
25,000	137	143	140	
5,000	70	76	73	
PBST	15	14	14.5	
